# The delivery of heavy menstrual bleeding services in England and Wales after publication of national guidelines: a survey of hospitals

**DOI:** 10.1186/1472-6963-13-491

**Published:** 2013-11-25

**Authors:** Suzanne M Cox, David Cromwell, Tahir Mahmood, Allan Templeton, Benedetta La Corte, Jan van der Meulen

**Affiliations:** 1 Center for Healthcare Studies, Northwestern University, 420 E Superior St, 10th Floor, Chicago, IL 60611, USA; 2 Department of Health Services Research and Policy, London School of Hygiene and Tropical Medicine, 15-17 Tavistock Place, London WC1H 9SH, UK; 3 Office for Research and Clinical Audit, Lindsay Stewart R&D Centre, Royal College of Obstetricians and Gynaecologists, 27 Sussex Place, Regent’s Park, London NW1 4RG, UK

**Keywords:** Heavy menstrual bleeding, Menorrhagia, Gynaecology, Health services, National guidelines

## Abstract

**Background:**

In 2007–2008, two UK-based organisations, the National Institute for Health and Clinical Excellence and the Royal College of Obstetricians and Gynaecologists, published guidelines for the management of care and organisation of outpatient services for women with heavy menstrual bleeding (HMB). In 2010, this study was conducted to provide an update on guideline-related services provided in England and Wales, and whether they are consistent with national clinical guidelines two to three years after publication.

**Methods:**

An organisational survey of outpatient gynaecology clinics was conducted of 221 hospitals in 154 acute National Health Service (NHS) trusts in England and Wales. A questionnaire was distributed to all hospitals to examine provision of diagnostic and therapeutic services in outpatient settings. Descriptive statistics were used to summarize results.

**Results:**

The response rate was 100%. For diagnosis, 80% of hospitals had ultrasound, 87% had hysteroscopy, and 98% had endometrial biopsy available. Overall, 76% of hospitals provided an information leaflet, 8% referred patients to a website for information, and 20% did not provide any written information. A dedicated menstrual bleeding clinic was present in 38% of hospitals. Only 30% of hospitals reported that they have a local written protocol regarding the care and management of women with HMB.

**Conclusion:**

The majority of hospitals offer appropriate diagnostic and surgical services for women with HMB. However, local protocol development may not reflect the local services. It may be that hospitals are finding it difficult to summon resources to provide clinics for women with menstrual disorders.

## Background

Heavy menstrual bleeding (HMB) is estimated to affect approximately 5% to 30% of women of reproductive age. It is the fourth most common cause for referral to gynaecological services in the UK [1,2]. HMB is a condition that significantly impacts women’s perceived physical and mental health over time (health-related quality of life) [3,4]. Between 2004 and 2006, there were 51,664 women with HMB aged between 25 and 59 years who underwent either an endometrial ablation or hysterectomy in English National Health Service (NHS) trusts [5]. Among women with HMB, surgical rates across English regions (defined by Strategic Health Authorities, SHAs) ranged from 52 to 230 procedures per 100,000 women [5].

In 2007, the National Institute for Health and Clinical Excellence (NICE) published *Heavy Menstrual Bleeding*, a national guideline on the management of HMB [6]. In 2008, the Royal College of Obstetricians and Gynaecologists (RCOG) also issued HMB-specific recommendations in their *Standards of Gynaecology*[7]. Despite this, significant regional variation in surgical treatment rates did not diminish between 2006 and 2009 [8].

The guidelines make various recommendations about the organisation of outpatient gynaecology services for women with HMB. These recommendations cover structural issues such as the creation of dedicated one-stop menstrual bleeding clinics, which include diagnostic facilities within the clinic, to the provision of HMB-specific health and treatment information to patients.

One potential explanation for the regional differences in surgical treatment rates of women with HMB was the variation in the organisation of gynaecological outpatient services and the degree to which guideline recommendations had been adopted. We therefore conducted an organisational survey of outpatient services for HMB in NHS hospitals in England and Wales, which was part of a larger ongoing project commissioned by the Healthcare Quality Improvement Partnership as part of the National Clinical Audit and Patient Outcomes Programme [9,10]. The organisational survey reported here examined the local provision of diagnostic and therapeutic services, local protocols for the management of HMB and issues related to referral patterns from primary care.

## Methods

All 221 NHS hospitals of 154 acute NHS trusts in England and Wales providing secondary care through outpatient gynaecology departments during June to September 2010 were eligible to participate. The hospitals were identified from various sources including the RCOG database of Clinical Directors and the NHS service provider websites for England (nhs.uk) and Wales (wales.nhs.uk). In accordance with current guidelines on clinical audit, no ethical committee review was required for the conduct of this project.

The questionnaire included items on various aspects of the NICE and RCOG guidelines, and practical aspects of service provision (Table 1). In addition, the hospitals were asked to return a copy of any and all local guidelines or protocols regarding the care and management of women with HMB. The questionnaire was designed in consultation with an Expert Advisory Group consisting of healthcare consumers and clinical representatives from gynaecology, nursing, general practice, psychology, and health services research. The survey questionnaire was created in both a paper-based and a web-based format. Both were piloted at six hospitals across England and Wales and updated in response to the comments from these pilot sites.

**Table 1:** Questionnaire design for the organisational survey of gynaecological outpatient services for heavy menstrual bleeding

**Domain**	**Topic**
Services and care for women with Heavy Menstrual Bleeding (HMB)	• Local written protocol or guideline for HMB*
	◦ Local protocols derived from national guidelines should be in place for speedy and evidence-based management of heavy menstrual bleeding in primary care. (RCOG)
	• Dedicated menstrual bleeding clinic*
	◦ There should be a dedicated one-stop menstrual bleeding clinic with facilities within the clinic for diagnostic gynaecology, including hysteroscopy and ultrasound. (RCOG)
	• If yes, is the clinic ‘one-stop’ (i.e., a clinic designed only to see patients with menstrual bleeding issues)?*
	◦ There should be a dedicated one-stop menstrual bleeding clinic with facilities within the clinic for diagnostic gynaecology, including hysteroscopy and ultrasound. (RCOG)
	• Facilities available within the department
	• Investigations at first consultation
	• Surgical treatment options*
	◦ In women with HMB alone, with uterus no bigger than a 10-week pregnancy, endometrial ablation should be considered preferable to hysterectomy. (NICE)
Referral to secondary care	• Referral system in the local area*
	◦ Referral pathways between primary and secondary care should be agreed locally and reviewed annually. (RCOG)
	• Baseline investigations generally carried out in primary care
	• Treatment offered in primary care*
	◦ Adequate facilities and trained individuals should be available for the insertion of levonorgestrel-releasing intrauterine system (LNG-IUS) in the outpatient clinic and in primary care settings. (RCOG)
	• Proportion of women with no treatment in primary care*
	◦Adequate facilities and trained individuals should be available for the insertion of levonorgestrel-releasing intrauterine system (LNG-IUS) in the outpatient clinic and in primary care settings. (RCOG)
	• Reasons for referral to secondary care
	• Average waiting time from referral to appointment
	• Management options in secondary care
	• Direct GP referral to diagnostic procedures*
	◦ Guidelines should be in place for direct referral to imaging services from primary care. (RCOG)
Information for patients	• Written information for patients about HMB*
	◦ An information leaflet should be available that includes each treatment option for heavy menstrual bleeding, together with outcomes and complications. (RCOG)
	◦ A woman with HMB referred to specialist care should be given information before her outpatient appointment. The Institute’s information for patients (‘Understanding NICE guidance’) is available from http://www.nice.org.uk/CG044publicinfo (NICE)
	• Timing of information
	• Who provides it
Patient questionnaires	• Formal questionnaire to assess how HMB affects women*
	◦ For clinical purposes, HMB should be defined as excessive menstrual blood loss which interferes with the woman’s physical, emotional, social and material quality of life, and which can occur alone or in combination with other symptoms. Any interventions should aim to improve quality of life measures. (NICE)
	◦ The treatment should aim to improve quality of life rather than focusing on menstrual blood loss alone. (RCOG)
	• Timing of questionnaire
	• Who provides it
Departmental information	• Number of first appointments in clinic overall
	• Number of first appointments for HMB

*Relevant guideline in *Standards for Gynaecology,* RCOG 2008, or *Heavy Menstrual Bleeding,* NICE 2007.

Hospitals were approached through the Clinical Directors of Obstetrics and Gynaecology or the Clinical Audit Departments. The person nominated to complete the questionnaire received their preferred questionnaire format. Up to three reminders were sent at approximately three week intervals after the initial distribution, and respondents could contact the study team to answer queries.

All analyses used descriptive statistics to summarise responses to the survey.

## Results

All 221 hospitals returned the questionnaire (response rate of 100%). Data completeness was very high with only 1-3% missing values for the questions reported here.

### Availability of hospital facilities

The majority of hospitals reported having access to the principal diagnostic facilities within their department to investigate patients with HMB. Overall, 98% (216/221) had access to endometrial biopsy, 80% (177/221) had ultrasound, and 87% (193/221) had hysteroscopy. In addition, 95% of hospitals (210/221) reported that they had “day care diagnosis” available, which combines inpatient-based hysteroscopy with endometrial biopsy. 38% of hospitals (84/219) reported they ran a dedicated menstrual bleeding clinic, of which 73 were described as a “one-stop” clinic that provided both diagnosis and treatment plan at the same appointment. Access to diagnostic facilities was slightly better in hospitals with these dedicated clinics (Table 2).

**Table 2:** Available facilities within gynaecology outpatient departments for diagnosis and treatment of heavy menstrual bleeding

**Facilities**	**No dedicated menstrual bleeding clinic**	**Dedicated menstrual bleeding clinic**
Ultrasound (trans-vaginal scanning in the clinic)	75.6% (102/135)	88.1% (74/84)
Hysteroscopy (outpatient based)	80.0% (108/135)	98.8% (83/84)
Endometrial biopsy (outpatient based)	96.3% (130/135)	100.0% (84/84)
Day care diagnosis (inpatient-based) hysteroscopy plus endometrial biopsy	95.6% (129/135)	94.0% (79/84)

All hospitals reported that endometrial ablation and hysterectomy were available surgical options. One or more second-generation ablation techniques were offered by 94% (207/221) of hospitals, of which fluid filled thermal balloon ablation was the most commonly available. Over 70% (158/221) of hospitals still offered the first-generation roller ball ablation technique, but only 5% (11/221) of hospitals offered this technique as their only ablation option.

### Patient management within outpatient departments

Hospitals were asked what investigations are considered at the initial consultation in their clinic for women with HMB being referred for the first time. In general, the responses followed the national recommendations (Table 3) [1,2]. An abdominal and pelvic examination was considered ‘mostly’ or ‘always’ by 99% (219/221) of hospitals, while an objective measurement of blood loss was ‘never’ or ‘rarely’ considered by 82% (181/220) of hospitals.

**Table 3:** Investigations considered at first outpatient gynaecology visit for women with heavy menstrual bleeding

	**Always(%)**	**Mostly(%)**	**Sometimes(%)**	**Rarely(%)**	**Never(%)**	**Responses(n)**
Abdominal and pelvic examination	83.7	15.4	0.9	0.0	0.0	221
Full blood count test	24.3	36.9	32.7	6.1	0.0	214
Ultrasound and other imaging	29.4	41.2	27.6	1.4	0.5	221
Pathology (e.g., endometrial biopsy)	8.7	42.0	47.0	1.8	0.5	219
Objective method of assessing blood loss	5.0	7.7	5.0	28.2	54.1	220

Availability of patient information varied among responding hospitals, as 76% (165/217) provided an information leaflet, while 8% (18/217) referred patients to a website for information. 20% (43/217) of hospitals did not provide written information.

Only 6% (13/221) of hospitals reported that they formally assess how HMB affects women’s lives using a quality of life instrument. The questionnaire could be sent prior to the first visit, or completed during or after the first visit.

Overall, 30% (64/214) of hospitals reported that they have a written local protocol on the management of women with HMB.

### Treatment in primary care and reasons for referral

Respondents reported on the investigations that had typically been performed in primary care before women were referred to their secondary care clinics (Table 4). Full blood count was the most commonly reported investigation in primary care with 94% (207/220) of hospitals reporting that this was ‘always,’ ‘mostly’ or ‘sometimes’ performed. 99% of hospitals (218/220) responded that General Practitioners (GPs) in their area could refer directly to imaging services. It was less common for GPs to be able to refer directly to pathology (in 42% of hospitals [94/220]) and other diagnostic procedures (in 22% of hospitals [48/220]).

**Table 4:** Primary care investigations for heavy menstrual bleeding before referral to outpatient gynaecology (reported by hospital)

	**Always(%)**	**Mostly(%)**	**Sometimes(%)**	**Rarely(%)**	**Never(%)**	**Don’t know(%)**	**Responses(n)**
Full blood count	3.6	48.2	42.3	5.9	0.0	0.0	220
Ultrasound	2.3	18.6	73.5	5.6	0.0	0.0	215
Thyroid function test	0.0	6.4	58.6	31.4	3.2	0.5	220
Hormonal assessment	0.0	4.6	51.6	40.1	3.7	0.0	217
Liver function test	0.0	0.5	8.1	56.9	34.0	0.5	209

There were 83% (181/218) of hospitals that reported over half of their patients had received some treatment in primary care, with tranexamic or mefanamic acid being most common (Table 5). Nonetheless, 17% of hospitals (37/218) reported that most or almost all of their patients did not receive any treatment in primary care.

**Table 5:** Primary care treatments prior to referral to an outpatient gynaecology department (reported by hospital)

**Treatment**	**Always(%)**	**Mostly(%)**	**Sometimes(%)**	**Rarely(%)**	**Never(%)**	**Responses(n)**
Tranexamic acid	1.4	36.4	60.5	1.8	0.0	220
Trial of treatment with mefenamic acid	2.3	28.2	65.5	4.1	0.0	220
Oral progestogens	0.5	12.4	68.7	18.0	0.5	217
Combined oral contraceptives (COCs)	0.9	6.4	77.2	15.1	0.5	219
Levonorgestrel-releasing intrauterine system (LNG-IUS) (e.g., Mirena)	0.0	4.6	67.0	24.8	3.7	218
Injected long-acting progestogens	0.0	1.9	47.4	48.8	1.9	215
Self-treatment	1.9	3.2	31.2	54.1	9.6	157

Hospitals were also asked the most common reasons why patients with HMB would be referred for the first time to their outpatient department. ‘Failure to respond to medical treatment in primary care’ was the most common reason (98% [214/219] responded this was ‘always,’ ‘mostly’ or ‘sometimes’ the case). ‘Patient was seeking a definite treatment such as hysterectomy’ was also common (94% [205/218] responded this was ‘always,’ ‘mostly’ or ‘sometimes’ the case). ‘Patient requesting referral to a specialist’ was slightly less common (86% [180/210]).

## Discussion

The organisational survey presented here sought to answer the question as to whether outpatient care for women with HMB currently met national guidelines, which had been developed by NICE and RCOG in response to “a lack of consensus about the best form of management of this condition” [6,7,11]. This organisational survey is the first step of a larger project that will collect information on the patient-reported outcomes of care for women with HMB in the first year after their first outpatient visit at NHS hospitals in England and Wales [9,10].

To adequately provide a national picture, the extent of coverage is critical for any national survey [12]. While we acknowledge that some specialised care for women with HMB may be provided by specialty primary care clinics or other women’s health clinics provided by the private sector, this national survey comprehensively describes the organisation of outpatient care in the NHS across England and Wales.

A limitation of the study design is the difficulty in translating national guidelines into survey questions. Many of the guidelines’ recommendations are very specific in the relationship between symptomology and resultant treatment options. Therefore, the organisational survey was limited to general issues such as the availability of diagnostic equipment, the availability of various treatment options, and referral patterns from primary to secondary care. In addition, for some survey items, yes-no questions (e.g., Does your hospital have a one-stop clinic?) rather than the implementation rates (e.g., What percentage of patients are able to access a one-stop clinic?) were asked. This limitation in precision of measurement was part of the design strategy wherein ease and accuracy of question completion was prioritised. The result allowed for measurement of whether hospitals had at least met the minimum level of the recommendation (e.g., setting up a one-stop clinic).

While an in-person audit of available services would have been an ideal way to measure adherence to recommendations, cost constraints limited the study to a self-report survey design. Whenever possible, reliability of responses was verified by asking Clinical Directors to oversee the completion of the survey; by engaging local Clinical Audit Departments; and by clarifying survey responses with hospital staff.

In their standards, the RCOG emphasises that “every organisation should clearly set out specific requirements relating to the management of excessive menstrual blood loss which interferes with a woman’s physical, social, emotional and material quality of life.” Hospitals were therefore asked whether their hospital had a local, written protocol. Surprisingly, less than one in three hospitals reported that they had such a protocol.

Another recommendation of the RCOG is that “there should be a dedicated one-stop menstrual bleeding clinic with facilities within the clinic for diagnostic gynaecology, including hysteroscopy and ultrasound.” This recommendation is aimed at streamlining services and reducing the burden on both health professionals’ and patients’ time. Again, only about one in three hospitals reported that this type of clinic was available. A similar proportion reported having a dedicated menstrual disorders clinic. This kind of clinic could be very beneficial to patients’ experience of care.

One in five hospitals reported that they do not provide an informational leaflet nor refer patients to a website. This is a remarkable observation as high-quality informational materials about HMB and its treatment options is readily available (see for example http://guidance.nice.org.uk/CG44/PublicInfo/doc/English).

Encouragingly, many of the diagnostic and therapeutic recommendations in the national guidelines are being met by most hospitals. For example, almost all hospitals (99%) reported that GPs in their area could refer directly to imaging services. Similarly, almost all hospitals (94%) offer one or more second-generation ablation techniques.

## Conclusions

The national survey of hospitals in England and Wales showed that basic care provision for women with HMB is being met through adequate levels of diagnostic and treatment facilities. However, key systems such as local protocols (which put in place agreements regarding referral and management of patients) and one-stop menstrual disorders clinics (which would streamline care and improve the care experience of patients with HMB) were reported by a minority of hospitals.

The National HMB Audit, a prospective national clinical audit collecting information reported by women who visit the outpatient gynaecology department of NHS hospitals in England and Wales for the first time is currently being carried out (http://www.rcog.org.uk/orca/audit). This audit aims to assess whether care and management of women with HMB can be further improved. It will provide key information on the impact that variations of service provision have on the outcomes of secondary care in terms of symptom severity and quality of life.

## Abbreviations

HMB: Heavy menstrual bleeding; GP: General practitioners; NHS: National health service; NICE: National institute for health and clinical excellence; RCOG: Royal college of obstetricians and gynaecologists; SHA: Strategic health authority.

## Competing interests

We declare that we have no financial or non-financial competing interests.

## Authors’ contributions

SC, DC, TM, AT, and JVDM were responsible for the initial plan and study design. SC and BDLC prepared all instruments and collected all data. SC, DC, and BDLC conducted data analysis. All authors were responsible for interpretation of data. SC drafted the article, and other co-authors revised it critically for important intellectual content. All authors have approved the final version.

## Pre-publication history

The pre-publication history for this paper can be accessed here:

http://www.biomedcentral.com/1472-6963/13/491/prepub
